# Aberrant static and dynamic brain functional topological organization in the differentiation of myelin oligodendrocyte glycoprotein antibody-seropositive optic neuritis from seronegative optic neuritis

**DOI:** 10.3389/fnins.2025.1627269

**Published:** 2025-09-12

**Authors:** Weiliang Qian, Yaru Sheng, Xilan Liu, Yan Sha, Ximing Wang, Ping Lu

**Affiliations:** ^1^Department of Radiology, The Affiliated Suzhou Hospital of Nanjing Medical University, Suzhou Municipal Hospital, Suzhou, China; ^2^Department of Radiology, Eye & ENT Hospital of Fudan University, Shanghai, China; ^3^Department of Radiology, Shanghai Children’s Medical Center, Dongfang, China; ^4^Department of Radiology, The First Affiliated Hospital of Soochow University, Suzhou, China

**Keywords:** functional network, topological organization, optic neuritis, myelin oligodendrocyte glycoprotein, magnetic resonance imaging

## Abstract

**Objective:**

An early and accurate diagnosis of myelin oligodendrocyte glycoprotein antibody seropositive optic neuritis (MOG-ON) versus seronegative-ON is critical for optimal management. We aimed to explore alterations in static and dynamic functional networks for differentiation by resting-state functional magnetic resonance imaging (RS-fMRI) with the graph theory method.

**Methods:**

RS-fMRI was performed on 53 patients (23 with MOG-ON and 30 with seronegative-ON) and 26 healthy controls (HCs). Graph theory analysis was used to investigate the topological properties of the functional networks. Receiver operating characteristic (ROC) curve analysis was also performed to determine their effectiveness in differential diagnosis.

**Results:**

With respect to static properties, the MOG-ON and seronegative-ON groups presented a spectrum of abnormalities in global and nodal properties compared with the HC group. Furthermore, compared with the seronegative-ON group, the MOG-ON group also presented with abnormal properties mostly located in the visual network (VN). With respect to dynamic properties, the MOG-ON and seronegative-ON groups presented with greater variances of global and nodal properties compared with the HC group. Importantly, the variances in several global and nodal properties were greater in the MOG-ON group. Compared with that in HCs, the subnetwork (24 nodes and 28 edges) in the MOG-ON patients was enhanced. For ROC analysis, the optimal diagnostic performance was obtained by combining static and dynamic approaches.

**Conclusion:**

In conclusion, abnormal topological organization of static and dynamic brain functional networks may help explore the neural mechanisms of ON in different phenotypes and serve as biomarkers for differentiation.

## Introduction

Optic neuritis (ON) is a common inflammatory demyelinating disease of the optic nerve that usually affects young adults and is typically characterized by varying degrees of acute visual loss, eye movement pain and visual field loss (De Lott et al., 2021; Petzold et al., 2022; Kraker and Chen, 2023). ON may occur in isolation or be associated with a variety of etiologies. Notably, ON may be the initial presentation of a central nervous system (CNS) demyelinating disease, such as multiple sclerosis (MS) or neuromyelitis optica (NMO) (De Lott et al., 2021; Kraker and Chen, 2023). Some atypical ON patients with seronegative aquaporin 4 (AQP4) still show bilateral attacks, relapses and steroid-dependent features. Awareness of the value of myelin oligodendrocyte glycoprotein (MOG) antibodies in the diagnosis and management of ON has increased in recent years (Zhao et al., 2018; Darakdjian et al., 2022; Petzold et al., 2022; Banwell et al., 2023; Moheb and Chen, 2023; Jeyakumar et al., 2024). MOG antibodies have already been implicated in demyelinated diseases, such as ON and acute disseminated encephalomyelitis (ADEM) (Li et al., 2020; Sechi et al., 2022; Banwell et al., 2023), and have recently been shown to be potential markers for differentiating atypical ON from other ON phenotypes (De Lott et al., 2021; Petzold et al., 2022; Kraker and Chen, 2023). Due to differences in immunopathogenesis, the therapeutic and follow-up strategies for MOG antibody-seropositive ON (MOG-ON) and seronegative (both MOG and AQP4) ON (seronegative-ON) patients need to be different for a favorable prognosis. Thus, early and accurate diagnosis of MOG-ON versus seronegative-ON is critical for optimal management (Petzold et al., 2022; Moheb and Chen, 2023). Antibody testing is recommended for atypical ON cases, particularly those with manifestations suggestive of NMO or MOG antibody disease (MOGAD). Delayed serological testing may hinder the prompt diagnosis of subtypes.

Clinically, cranial MRI is undoubtedly important in patients with ON. The role of optic nerve lesion length, extent of involvement and diffusion characteristics in differentiating different types of ON, such as MS-related ON, NMO-related ON and MOG-ON, has been reported (Lu et al., 2018; Winter and Chwalisz, 2021; Darakdjian et al., 2022; Carnero Contentti et al., 2023; Sarin et al., 2023; Jeyakumar et al., 2024). Because the intracranial visual cortex or network is the final processing part of the visual information transmitted from the retina and optic nerve, significant visual impairment may cause disturbances in the visual network. Therefore, studying changes in brain function networks may reveal commonalities and differences in the neural mechanisms of ON in different phenotypes.

Structural changes in the visual pathway have already been reported in ON and MOGAD (Tur et al., 2016; Bollo et al., 2024). Therefore, it would be interesting to study the potential functional changes caused by these structural changes, as this would improve our understanding of the mechanisms behind them. Currently, resting-state functional magnetic resonance imaging (RS-fMRI) is a paradigm that can probe spontaneous brain function without a task via blood oxygen level-dependent contrast (Barkhof et al., 2014). Spatially distributed networks of temporal synchrony can be detected, further characterizing brain function. Typically, functional brain network analysis via RS-fMRI has been used to reveal the physiological nature of interactions between functional brain regions (Luppi et al., 2024). In addition, resting-state functional connectivity and interactions are thought to be dynamic on the scale of several seconds, and time-varying brain graph performance is being investigated (Zhang et al., 2023; Laumann et al., 2024; Wang et al., 2024a; Zhou et al., 2024). Sliding window analysis, a common method that divides the time series of RS-fMRI data into several time windows based on an appropriate window size and step size, can be used to explore time-varying features by constructing a dynamic functional network (Wang et al., 2024a; Zhou et al., 2024). Graph theoretical analysis, which provides a powerful framework for characterizing topological properties in either static or dynamic brain functional connectomes, can be used to assess the properties of nodes (brain regions) and edges (connections between nodes) at both the local and global levels (Yu et al., 2018; Huang et al., 2019; Yu et al., 2022; Wang et al., 2024b). To date, various studies using the graph theory method based on static or dynamic functional networks have revealed altered topological properties in diseases associated with visual impairment (Huang et al., 2019; Wang et al., 2024a; Zhou et al., 2024); however, static and dynamic brain functional network research on MOG-ON is still lacking. Antibody testing can answer the question, ‘What is the cause?’ It is particularly effective at identifying specific immunological causes. More significantly, RS-fMRI can address a different question: ‘What will the outcome be?’ It can probe the functional integrity and plasticity of the entire visual system, measuring its capacity to process information following injury and providing an indication of brain function rather than merely serology. RS-fMRI can therefore serve as an important supplementary tool for serological testing.

Our study aimed to investigate the potential changes in static and dynamic functional connectomes in patients with MOG-ON and seronegative-ON using graph theoretical analysis based on RS-fMRI and to further evaluate its diagnostic value in differentiating MOG-ON from seronegative-ON. It may help us to better understand the neural mechanisms of different phenotypes of ON from a pathophysiological perspective.

## Materials and methods

### Subjects

The Institutional Review Board of our hospital approved this study. Prior to the addition of RS-fMRI to conventional orbital MRI, all patients were informed of the methods, purpose and potential risks of the study and provided written informed consent. During the period from June 2021 to February 2024, 53 patients who presented to our hospital with ON and had available serum MOG-Ab test results were enrolled in the study. Among the 53 patients with ON, 23 and 30 patients had MOG-ON and seronegative-ON, respectively. All patients had their diagnosis confirmed by a neuro-ophthalmologist. The criteria required for the diagnosis of ON and detailed study inclusion were as follows: (1) acute, newly onset visual loss with the presence or absence of pain with eye movements within the last month and/or unilateral or bilateral visual field defects; (2) a relative afferent pupillary defect (if the damage was unequal) or abnormal visual evoked potentials; and (3) no evidence of compressive, infectious, toxic, ischemic or hereditary etiologies. The exclusion criteria were as follows: (1) patients with a previous episode of ON, cranial or spinal demyelinating lesions; (2) patients who were not screened for MOG and AQP4 antibodies; and (3) patients with pathological myopia with severe refractive error or a history of ocular diseases (such as diabetic retinopathy or glaucoma) or neurodegenerative diseases (such as Parkinson’s or Alzheimer’s disease). After serum MOG and AQP4 antibody testing, ON patients were divided into MOG-ON and seronegative-ON (both MOG and AQP4 antibodies negative) groups. The healthy control (HC) group consisted of 26 healthy volunteers with no neurological or ophthalmological disorders who were matched to the ON group for age, sex and education.

### MRI protocol

RS-fMRI data were acquired using a 3.0 T MR system (Magnetom Prisma; Siemens Healthcare) equipped with a 64-channel head and neck coil. High-resolution structural T1-weighted images (T1WI) were acquired via the following parameters: repetition time (TR)/echo time (TE), 2200/2.48 ms; flip angle (FA), 8°; field of view (FOV), 230 × 230 mm; matrix, 256 × 256; thickness, 1 mm; no intersection gap; slices, 176; and scan acquisition time, 5 min 38 s. Functional images were acquired via the following parameters: TR/TE, 2000/30 ms; flip angle (FA), 90°; FOV, 220 × 220 mm; matrix, 64 × 64; thickness, 4 mm; slices, 35; 200 resting volumes; and acquisition time for scanning, 6 min 48 s. During the examination, all the subjects were asked to close their eyes and avoid any deliberate movements, not to think about anything in particular and not to fall asleep.

### Data preprocessing

RS-fMRI data were preprocessed using the Resting-State fMRI Data Analysis Toolkit plus V1.28 (RESTplus V1.28, http://www.restfmri.net/forum/restplus) via SPM12^1^ implemented in MATLAB (version R2022b, MathWorks, Inc., Natick, MA, United States). After discarding the first 10 time points of the functional images, slice timing correction and realignment for head motion correction were performed on the remaining 190 volumes. No subject had excessive head motion, as defined by a maximum translational movement of more than 2.0 mm or rotation of more than 2.0°. The individual structural T1WIs were then registered to the mean RS-fMRI data; the resulting aligned T1WIs were segmented via the Diffeomorphic Anatomical Registration Through Exponentiated Lie Algebra (DARTEL) toolbox to improve the spatial accuracy of RS-fMRI data normalization (Goto et al., 2013); and the data were spatially normalized to the Montreal Neurological Institute (MNI) space (resliced voxel size = 3 × 3 × 3 mm^3^). Linear detrending and nuisance covariate regression were then performed to regress out the effects of the 6 head motion parameters, white matter signals and cerebrospinal fluid (CSF) signals. A temporal bandpass filter (0.01–0.08 Hz) was also used to reduce the effects of low- and high-frequency physiological noise.

### Static network construction and analysis

The static networks were constructed using the GRETNA toolbox^2^ (Wang et al., 2015). The graph theoretical network analysis was based on two basic elements: nodes, which represent brain regions, and edges, which represent functional connections between regions. Definition of nodes: The Anatomical Automatic Labelling Atlas was used to parcellate the brain into 90 regions. Edges definition: The Pearson correlation coefficient of each pair of brain regions was calculated via the mean time series, and only the positive connections were considered. This process resulted in a 90 × 90 symmetric correlation matrix for each subject, and a Fisher z-transformation was then performed on all individual correlation matrices. The sparsity threshold for the calculation of graph theoretical properties was set between 6 and 38%, with an interval of 1%. Finally, the area under the curve (AUC) was calculated for this wide sparsity range to exclude the bias of a single sparse threshold.

The topological properties of each network were examined at both the global and local levels. The global properties included global efficiency (Eg), local efficiency (Eloc), the clustering coefficient (Cp), the characteristic path length (Lp), the normalized clustering coefficient (γ), the normalized characteristic path length (λ) and small-worldness (σ). Nodal properties included nodal efficiency (Ne), degree centrality (Dc), betweenness centrality (Bc), the nodal clustering coefficient (NCp), nodal local efficiency (NLe), and the nodal shortest path length (NLp).

### Dynamic network construction and analysis

The dynamic network was constructed using the DynamicBC toolbox^3^ via the sliding window approach (Liao et al., 2014). The window size and overlap were 30 TR and 0.97 (Wang et al., 2024b; Zhou et al., 2024), respectively, and the time series of the RS-fMRI data were divided into 161 windows. Based on the same sparsity range as the static network, we also calculated the AUC for the global and nodal topological properties in each time window. The variance of the AUCs between the 161 windows was then calculated for each subject to reflect the variability of the dynamic functional network over time.

### Ophthalmologic examination

The best-corrected visual acuity (BCVA) was examined with the international standard VA chart at a distance of 5 meters. Spectral-domain optical coherence tomography (SD-OCT) was used to measure the peripapillary retinal nerve fiber layer (pRNFL) with the RNFL 3.45 scan mode and to calculate the macular ganglion cell-inner plexiform layer (mGCIPL) within the central 6 mm diameter area of the macula. The OCT examination was performed within 4 weeks of symptom onset. The BCVA and OCT results that were recorded and used for statistical analysis were measurements taken from the ON eyes.

### Statistical analysis

Demographic and clinical data were analyzed via the chi-square test (*χ*^2^) and analysis of variance (ANOVA) in SPSS software (version 22.0, Chicago, IL). Network metrics were analyzed via GRETNA and BrainNet Viewer software^4^ to visualize the results. One-way analysis of covariance (ANCOVA) was performed to compare network topological properties among the three groups, with age, sex, education level and BCVA entered as covariates. The AUC of each network property and the variance of the AUCs among 161 windows were compared in the static and dynamic functional networks, and multiple comparisons were corrected via false discovery rate (FDR) correction (*p* < 0.05). A *post hoc t*-test was also used to identify statistically significant differences between two specific groups. To locate the altered functional connectivity between specific brain regions in ON patients, the network-based statistics (NBS) method^5^ was used for edge analysis (Zalesky et al., 2010). The primary cluster-defining threshold (*p* < 0.001) was used to define a set of suprathreshold edges between connected components (Zhou et al., 2024). The non-parametric permutation method with 5,000 permutations was then used to calculate the significance of each component, with age, sex, education level and BCVA entered as covariates. A corrected *p*-value was set at 0.05 for multiple comparisons. In addition, Pearson’s or Spearman’s rank correlation coefficients were calculated for indicators that differed from those of the HCs to examine the correlations of functional parameters with BCVA, OCT measurements and duration of disease in patients. Based on the statistically significant metrics obtained from the above steps, receiver operating characteristic (ROC) curve analyses were performed to assess the diagnostic performance of static and dynamic network changes and their combination in differentiating MOG-ON from seronegative-ON.

## Results

### Patient demographics

A total of 53 ON patients, 23 with MOG-ON and 30 with seronegative-ON, were recruited from the neuro-ophthalmology department of our hospital. The healthy volunteers were matched for age, sex and education to the ON patients. No significant differences in age, sex or education were found between the 3 groups or in disease duration, BCVA, pRNFL and mGCIPL between MOG-ON patients and seronegative-ON patients (all *p* > 0.05). MOG-ON patients tended to show more bilateral morbidity (30.4% vs. 10.0%, *p* = 0.062). The demographic and clinical characteristics are shown in Table 1.

**Table 1:** Demographic and clinical characteristics of ON patients and HCs.

Features	MOG-ON (*n* = 23)	Sneg-ON (*n* = 30)	HCs (*n* = 26)	*P*-value
Gender (female/male)	12/11	13/17	12/14	0.812
Age (years) (range)	32.1 ± 11.8 (20–63)	35.3 ± 10.6 (19–68)	33.9 ± 11.1 (20–61)	0.381
Education (years)	11.4 ± 4.8	12.1 ± 4.2	11.8 ± 3.9	0.687
Disease duration (days)	14.7 ± 6.3	15.7 ± 5.9	/	0.210
Bilateral	7 (30.4%)	3 (10.0%)	/	0.062
BCVA (at nadir)	0.25 ± 0.27	0.29 ± 0.25	0.93 ± 0.12	0.550^*^
pRNFL (μm)	100.5 ± 12.8	105.4 ± 10.7	/	0.217
mGCIPL (μm)	66.8 ± 11.4	70.1 ± 10.9	/	0.345
Serum MOG/AQP4 antibodies	+/−	−/−	/	/
Cranial or spinal demyelinating lesions (MRI scan of the first episode of ON)	None	None	/	/
Final diagnosis	MOGAD	Idiopathic	/	/

Sneg-ON, seronegative-ON. *p-value in the comparison between MOG-ON and Sneg-ON; pRNFL, peripapillary retinal nerve fiber layer; mGCIPL, macular ganglion cell-inner plexiform layer.

### Static network topology analysis

**Figure 1:**
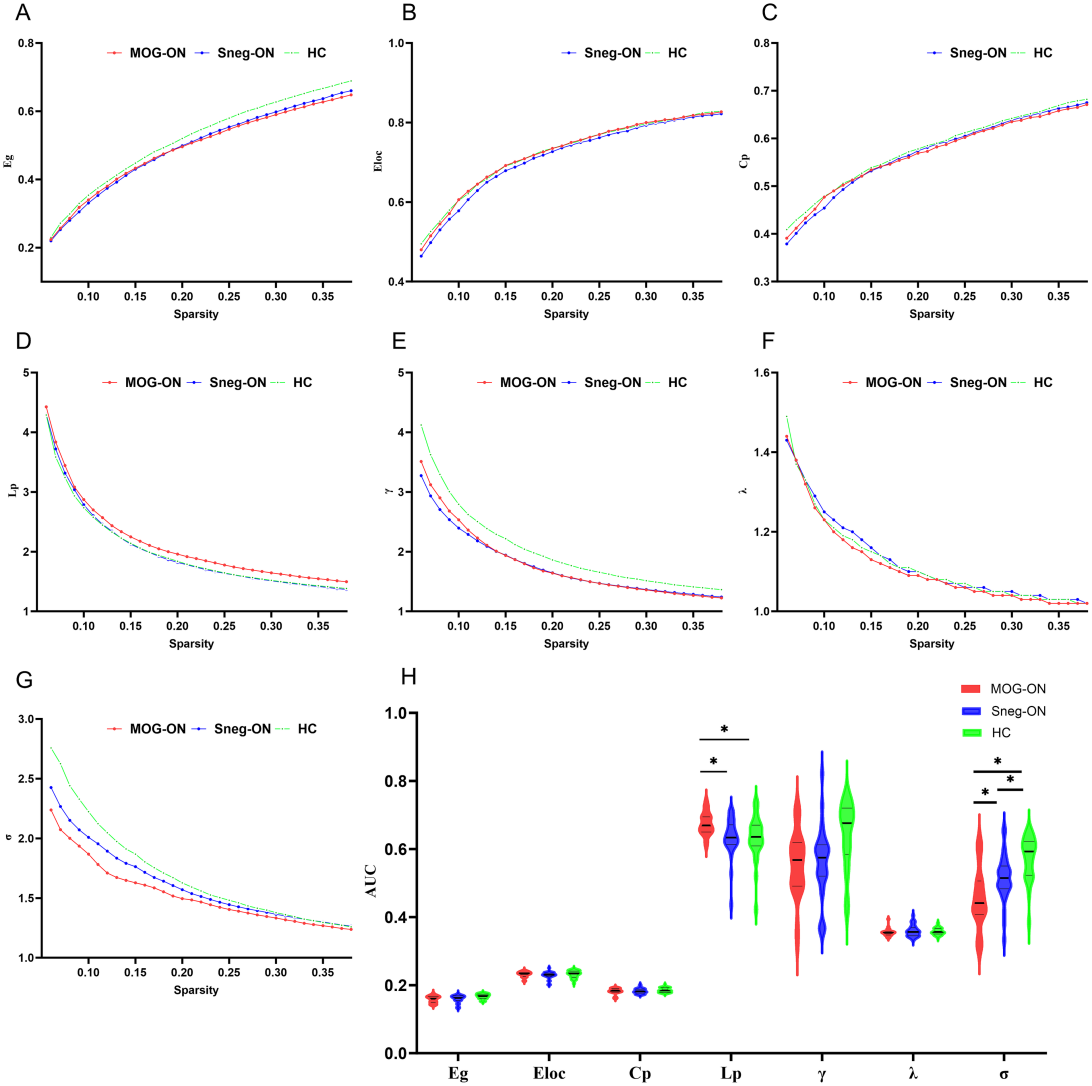
Global topological properties of the static functional network in MOG-ON patients, seronegative-ON patients and HCs. **(A–G)** Global properties among the 3 groups over the defined range of the sparsity threshold (0.06–0.38). **(H)** Comparisons of the AUCs of the global properties over the whole sparsity range. Significant differences between groups are marked with “*”.

In terms of global metrics, compared with HCs, both MOG-ON and seronegative ON patients presented significantly decreased *σ* values, and only MOG-ON patients presented increased Lp values (all *p* < 0.05). Compared with seronegative-ON patients, MOG-ON patients presented significantly increased Lp and decreased σ values. The Eg, Eloc, CP, *γ* or *λ* did not significantly differ among the 3 groups (Table 2 and Figure 1).

**Table 2:** Altered global properties of static and dynamic functional networks among MOG-ON patients, seronegative-ON patients, and HCs.

Parameters	ANCOVA *P* (F)	*Post hoc* (↑/↓/–)		
		MOG-ON vs. Sneg-ON	MOG-ON vs. HCs	Sneg-ON vs. HCs
Eg	0.099 (2.387)	–	–	–
Eloc	0.682 (0.384)	–	–	–
Cp	0.305 (1.206)	–	–	–
Lp	0.015 (4.481)	↑	↑	–
γ	0.234 (1.479)	–	–	–
λ	0.436 (0.839)	–	–	–
σ	<0.001 (9.312)	↓	↓	↓
Eg_var	0.003 (6.300)	–	↑	↑
Eloc_var	<0.001 (8.955)	↑	↑	–
Cp_var	0.484 (0.733)	–	–	–
Lp_var	0.001 (8.031)	↑	↑	↑
γ_var	0.009 (5.930)	↑	–	–
λ_var	0.761 (0.274)	–	–	–
σ_var	<0.001 (9.907)	↑	↑	↑

Eg, global properties included global efficiency; Eloc, local efficiency; Cp, clustering coefficient; Lp, characteristic path length; γ, normalized clustering coefficient; λ, normalized characteristic path length; σ, small-worldness; Eg_var., Eloc_var., Cp_var., Lp_var., γ_var., λ_var., σ_var., variance of the corresponding parameters; Sneg-ON, seronegative-ON.

*Post hoc*, ↑, ↓, − represent a significant increase and decrease in the former compared with the latter and no significant difference, respectively.

**Figure 2:**
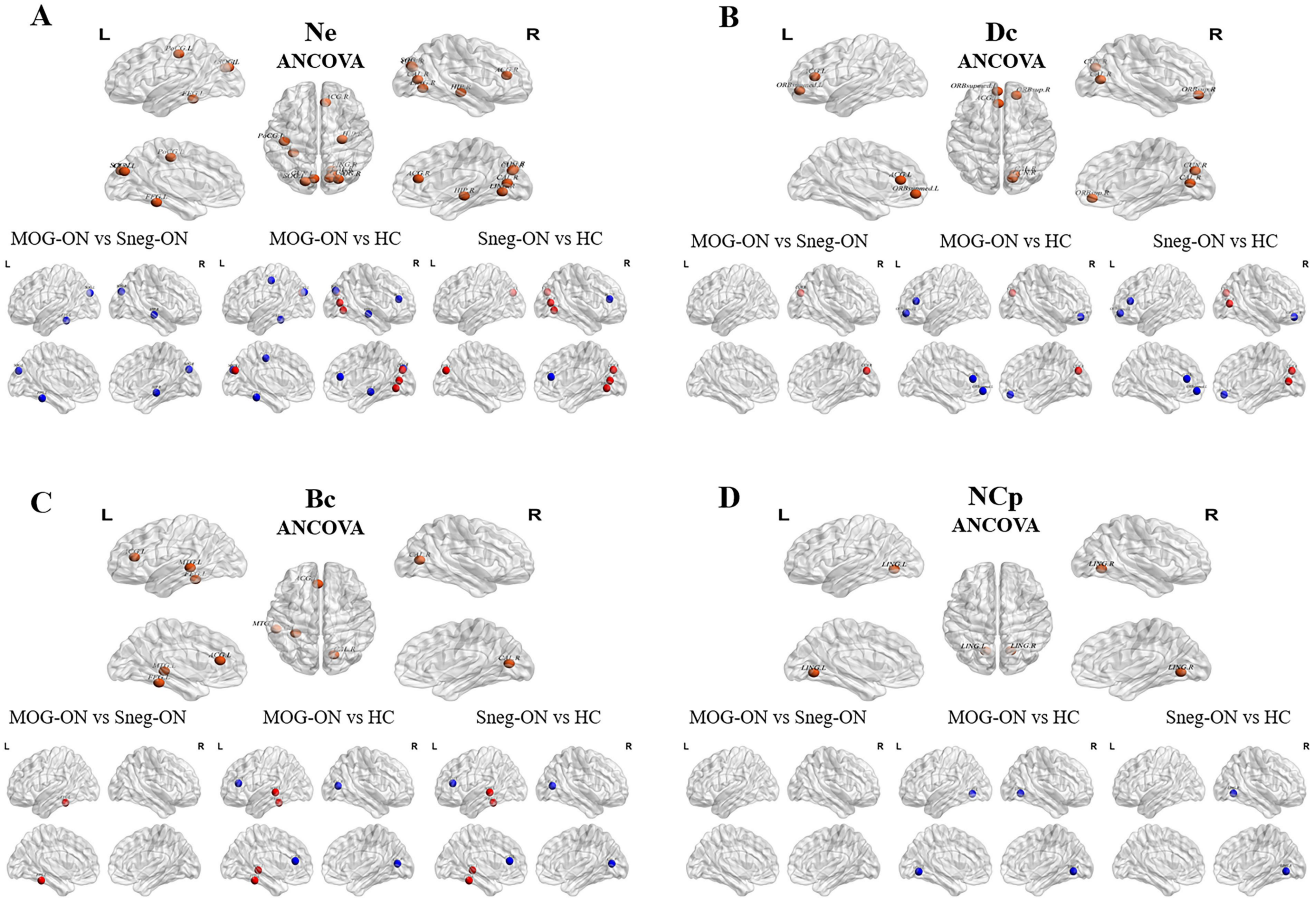
Nodes with significantly different nodal properties in MOG-ON patients, seronegative-ON patients and HCs. **(A–D)** Nodes with altered Ne, Dc, Bc, and NCp values among the 3 groups are depicted in orange. In the pairwise comparisons, nodes with increased or decreased properties (the former compared with the latter) are depicted in red and blue, respectively.

Compared with HCs, MOG-ON and seronegative-ON patients presented altered Ne, Dc, Bc and NCp values in several brain regions; these changes were mostly located in the visual network (VN) and parts of the frontoparietal and temporal lobes (all *p* < 0.05; FDR corrected). The specific changes were increased Ne, Dc and Bc values in the calcarine fissure and surrounding cortex (CAL), cuneus (CUN), lingual gyrus (LING), fusiform gyrus (FFG) and middle temporal gyrus (MTG) and decreased Ne, Dc, Bc and NCp values in the orbital part of the superior frontal gyrus (ORBsup), the medial orbital part of the superior frontal gyrus (ORBsupmed), anterior cingulate and paracingulate gyrus (ACG), hippocampus (HIP), CAL, LING, superior occipital gyrus (SOG), FFG and postcentral gyrus (PoCG). Notably, compared with seronegative-ON patients, MOG-ON patients exhibited significantly decreased Ne values in the HIP, SOG and FFG and increased Dc and Bc values in the CUN and FFG (Table 3 and Figure 2).

**Table 3:** Altered local properties of the static functional network among MOG-ON patients, seronegative-ON patients, and HCs.

Parameters	ANCOVA *P* (F)	Post hoc (↑/↓/–)		
		MOG-ON vs. Sneg-ON	MOG-ON vs. HCs	Sneg-ON vs. HCs
Ne				
ACG. R	0.005 (5.965)	–	↓	↓
HIP. R	0.006 (5.567)	↓	↓	–
CAL. R	<0.001 (9.580)	–	↑	↑
CUN. L	0.001 (8.034)	–	↑	↑
CUN. R	0.006 (5.501)	–	↑	↑
LING. R	<0.001 (9.170)	–	↑	↑
SOG. L	<0.001 (11.432)	↓	↓	–
SOG. R	0.009 (5.040)	↓	↓	–
FFG. L	0.001 (7.754)	↓	↓	–
PoCG. L	0.005 (5.771)	–	↓	–
Dc				
ORBsup. R	0.001 (7.949)	–	↓	↓
ORBsupmed. L	0.006 (5.430)	–	↓	↓
ACG. L	0.005 (5.841)	–	↓	↓
CAL. R	0.008 (5.166)	–	–	↑
CUN. R	0.001 (7.842)	↑	↑	↑
Bc				
ACG. L	0.005 (5.772)	–	↓	↓
CAL. R	0.001 (8.013)	–	↓	↓
FFG. L	0.006 (5.439)	↑	↑	↑
MTG. L	0.008 (5.231)	–	↑	↑
NCp				
LING. L	<0.001 (10.120)	–	↓	–
LING. R	0.001 (8.511)	–	↓	↓

Ne, nodal efficiency; Dc, degree centrality; Bc, betweenness centrality; NCp nodal clustering coefficient; ACG, anterior cingulate and paracingulate gyrus; HIP, hippocampus; CAL, calcarine fissure and surrounding cortex; CUN, cuneus; LING, lingual gyrus; SOG, superior occipital gyrus; FFG, fusiform gyrus; PoCG, postcentral gyrus; ORBsup, orbital part of the superior frontal gyrus; ORBsupmed, medial orbital part of the superior frontal gyrus; MTG, middle temporal gyrus; R, right; L, left; Sneg-ON, seronegative-ON.

Multiple comparisons were corrected via FDR correction (*P* < 0.05). *Post hoc*, ↑, ↓, − represent a significant increase and decrease in the former compared with the latter and no significant difference, respectively.

### Dynamic network topology analysis

**Figure 3:**
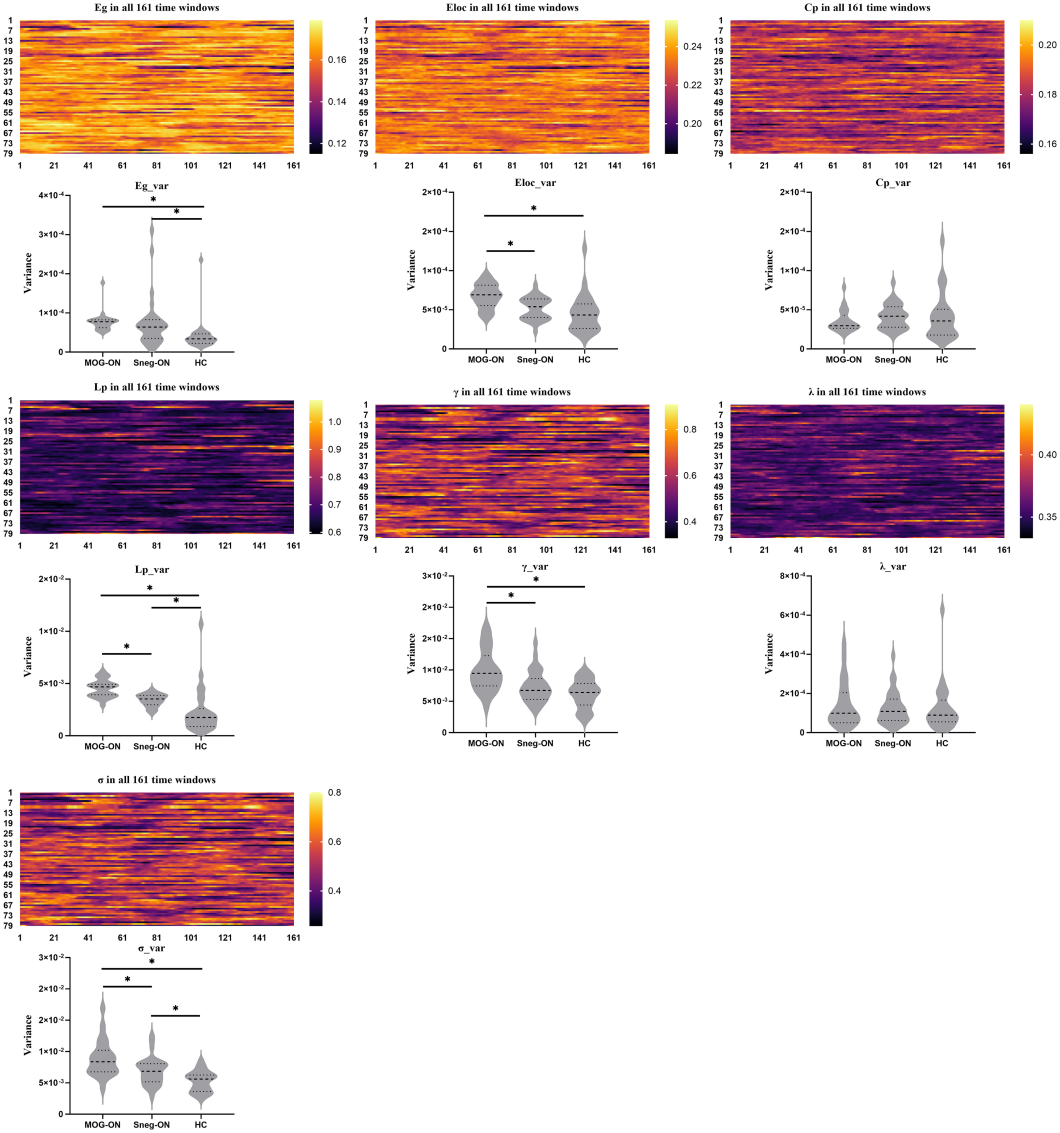
AUCs of the global properties for all 79 individuals over 161 time windows are presented as heatmaps. The variance of the AUCs between the 161 windows was calculated for each subject to reflect the variability of the global properties over time (Eg_var., Eloc_var., Cp_var., Lp_var., *γ*_var., *λ*_var., *σ*_var). Comparisons of the variance are presented as violin figures. Significant differences between groups are marked with “*”.

Compared with HCs, both MOG-ON and seronegative-ON patients had greater variances in Eg, Eloc, Lp, and σ (Eg_var., Eloc_var., Lp_var., σ_var) among the 161 time windows (all *p* < 0.05); furthermore, compared with seronegative-ON patients, MOG-ON patients had greater Eloc_var., Lp_var., γ_var, and σ_var. The variances of CP and λ (Cp_var., λ_var) did not significantly differ among the 3 groups (Table 2 and Figure 3).

**Figure 4:**
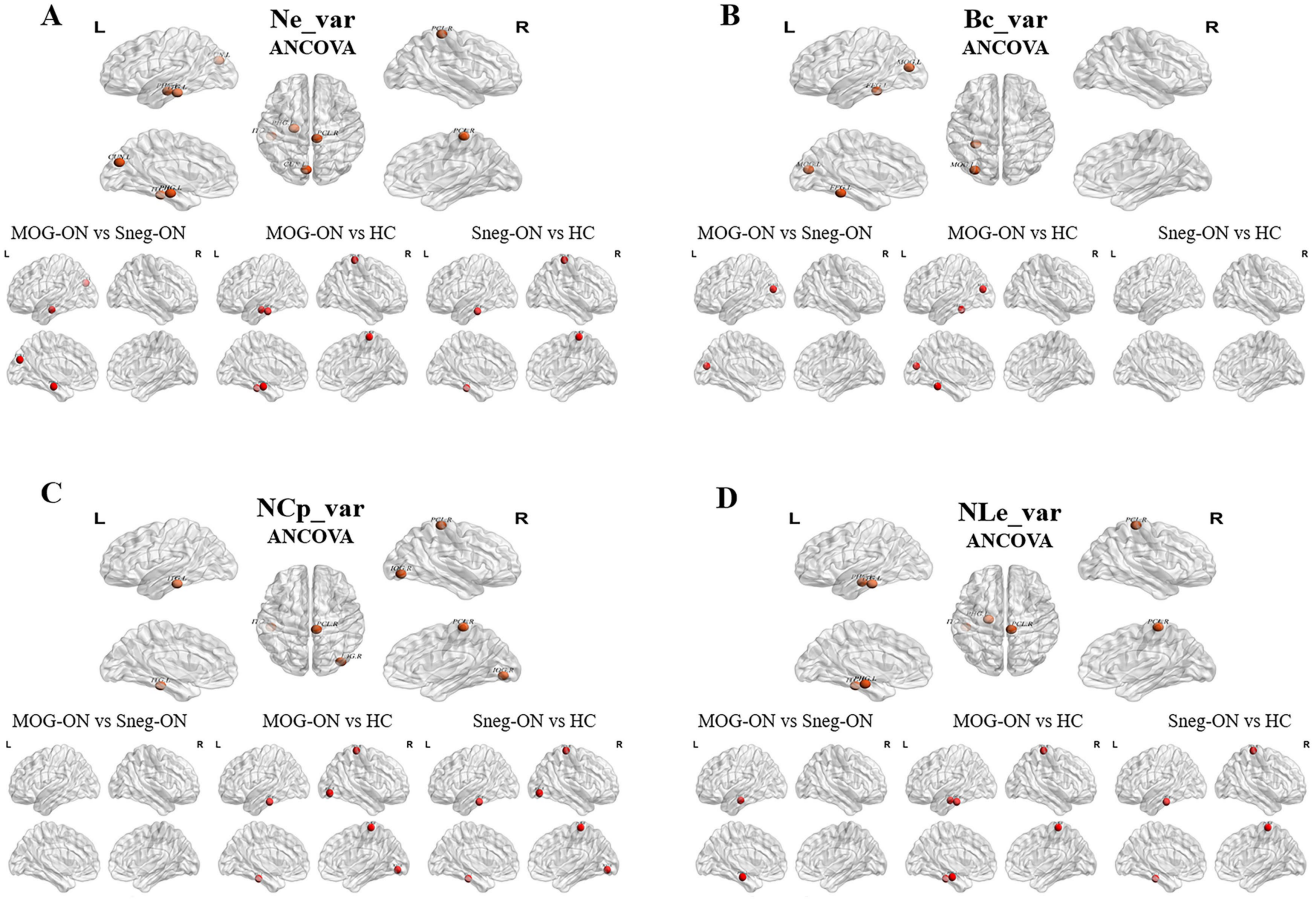
Variances in nodal properties with significant differences among MOG-ON patients, seronegative-ON patients and HCs. **(A–D)** Nodes with statistically altered Ne_var., Bc_var., NCp_var., and NLe_var values among the 3 groups are depicted in orange. In the pairwise comparisons, nodes with increased properties (the former compared with the latter) are depicted in red.

Compared with HCs, MOG-ON and seronegative-ON patients presented significantly increased variances in Ne, Bc, NCp and NLe (Ne_var., Bc_var., NCp_var, and NLe_var) in the parahippocampal gyrus (PHG), CUN, middle occipital gyruss (MOG), FFG, inferior occipital gyrus (IOG), paracentral lobule (PCL) and inferior temporal gyrus (ITG) (all p < 0. 05; FDR corrected); furthermore, compared with seronegative-ON patients, MOG-ON patients had greater Ne_var., Bc_var, and NLe_var values in the PHG, CUN and MOG (Table 4 and Figure 4).

**Table 4:** Altered local properties of the dynamic functional network among MOG-ON patients, seronegative-ON patients, and HCs.

Parameters	ANCOVA *P* (F)	Post hoc (↑/↓/–)		
		MOG-ON vs. Sneg-ON	MOG-ON vs. HCs	Sneg-ON vs. HCs
Ne_var				
PHG. L	0.001 (8.121)	↑	↑	–
CUN. L	0.001 (7.927)	↑	–	–
PCL. R	0.002 (6.604)	–	↑	↑
ITG. L	0.001 (7.914)	–	↑	↑
Bc_var				
MOG. L	<0.001 (12.024)	↑	↑	–
FFG. L	0.002 (6.776)	–	↑	–
NCp_var				
IOG. R	0.001 (7.611)	–	↑	↑
PCL. R	<0.001 (11.194)	–	↑	↑
ITG. L	0.001 (8.205)	–	↑	↑
NLe_var				
PHG. L	0.003 (6.266)	↑	↑	–
PCL. R	<0.001 (10.759)	–	↑	↑
ITG. L	0.009 (5.061)	–	↑	↑

Ne_var., Bc_var., NCp_var., NLe_var., variance of the corresponding parameters; PHG, parahippocampal gyrus; CUN, cuneus; PCL, paracentral lobule; ITG, inferior temporal gyrus; MOG, middle occipital gyrus; FFG, fusiform gyrus; IOG, inferior occipital gyrus; R, right; L, left; Sneg-ON, seronegative-ON.

Multiple comparisons were corrected via FDR correction (*P* < 0.05). *Post hoc*, ↑, ↓, − represent a significant increase and decrease in the former compared with the latter and no significant difference, respectively.

### Altered functional network connectivity

**Figure 5:**
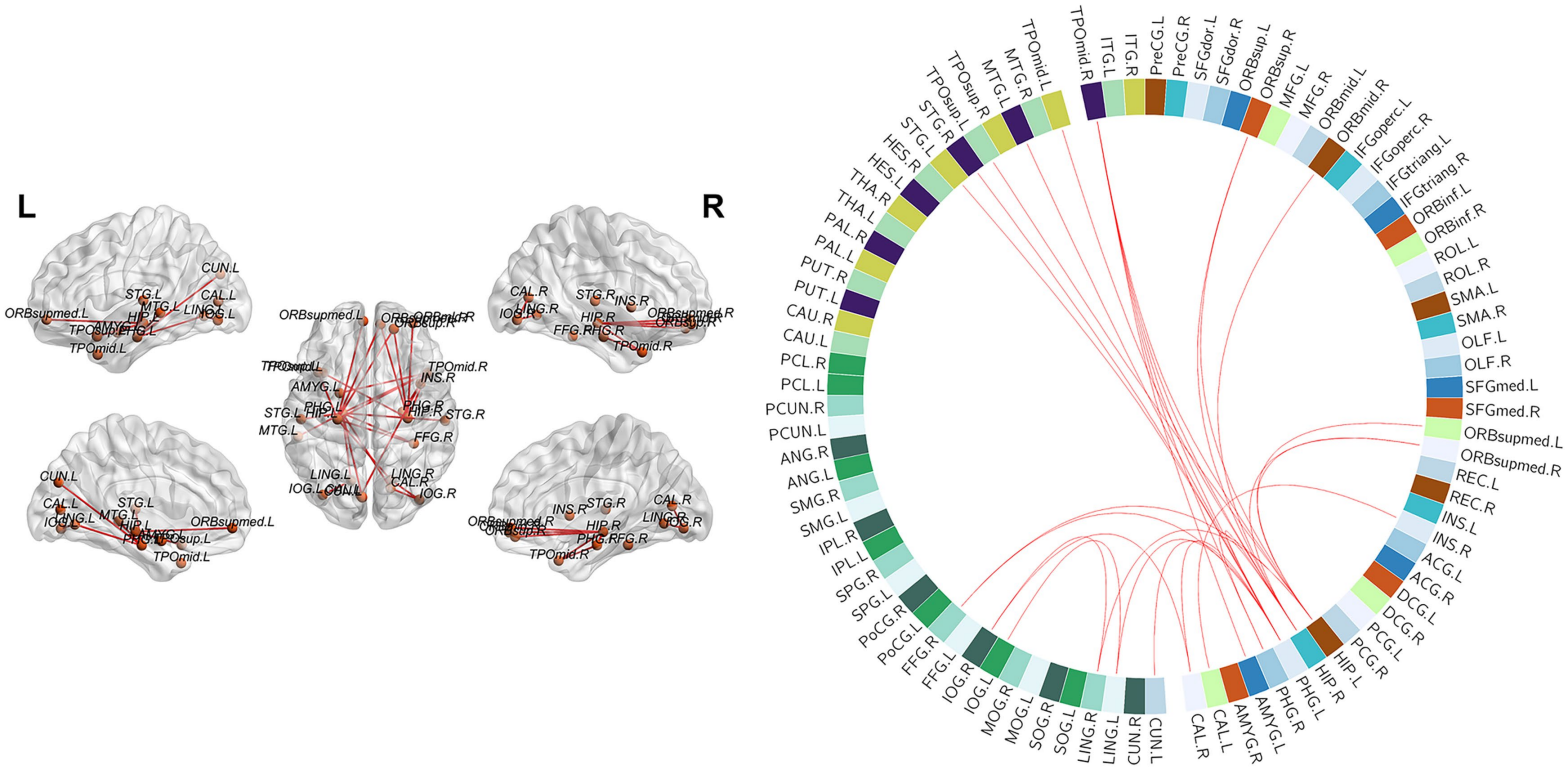
NBS analysis identified an enhanced subnetwork (24 nodes and 28 edges) in MOG-ON patients compared with HCs. The included nodes and edges were mostly located between the VN and the temporal lobe, and a small part was located between the frontal and temporal lobes. All nodes involved in the connections of this subnetwork: ORBsup, orbital part of the superior frontal gyrus; ORBmid, orbital part of the middle frontal gyrus; ORBsupmed, medial orbital part of the superior frontal gyrus; INS, insula; HIP, hippocampus; PHG, parahippocampal gyrus; AMYG, amygdala; CAL, calcarine fissure and surrounding cortex; CUN, cuneus; LING, lingual gyrus; IOG, inferior occipital gyrus; FFG, fusiform gyrus; STG, superior temporal gyrus; TPOsup, superior temporal gyrus of the temporal pole; MTG, middle temporal gyrus; TPOmid, middle temporal gyrus of the temporal pole.

The NBS analysis revealed a significantly enhanced subnetwork (24 nodes and 28 edges) in the MOG-ON group compared to the HC group (*p* < 0.05; threshold *T* = 3.273). The included nodes (brain regions) and edges (functional connections) were mostly located between the VN and the temporal lobe, with a small part located between the frontal and temporal lobes. Unfortunately, no significant differences were identified between MOG-ON patients and seronegative-ON patients or between seronegative-ON patients and HCs. The specific nodes and connections are shown in Figure 5.

### Correlation analysis

No significant correlation was found between functional topological organization metrics and BCVA, OCT measurements or disease duration in the patient groups.

### ROC curve analysis

**Figure 6:**
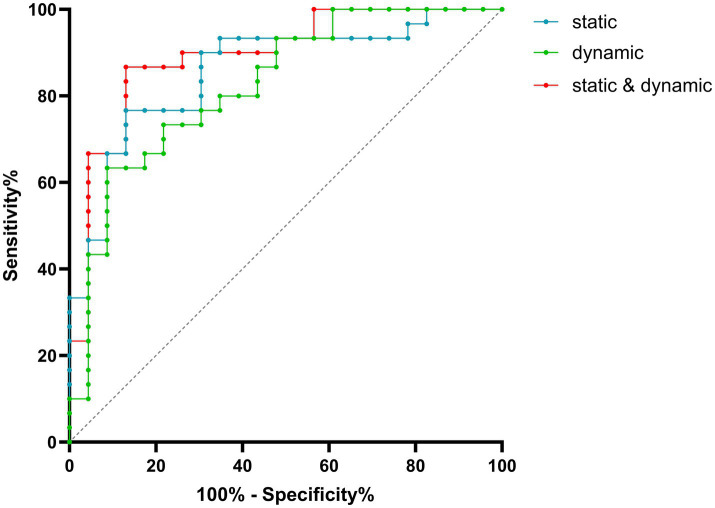
Results from the ROC curves. The AUCs of the static model, dynamic model and integrated model were 0.838, 0.797 and 0.873, respectively.

Based on the abnormal functional topological organization metrics, ROC curve analysis was performed to assess the performance of the models in differentiating MOG-ON from seronegative-ON. The static functional network diagnostic model (Model 1) was constructed via the combination of global and nodal properties, and the dynamic functional network model (Model 2) was based on the combination of the variance of global and nodal properties. The integrated model (Model 3) combined the approaches of Models 1 and 2. The AUCs of Models 1, 2 and 3 were 0.838, 0.797 and 0.873, respectively (Figure 6).

## Discussion

Studies on imaging techniques that can effectively differentiate MOG-ON and seronegative-ON are scarce, but this distinction is important for clinical practice. Previous studies (Lu et al., 2018; Winter and Chwalisz, 2021; Darakdjian et al., 2022) using conventional MRI have described some features of optic nerve lesions in different subtypes of ON but have not investigated the neural network changes underlying visual impairment. We hypothesize that the functional brain network connectomes of MOG-ON and seronegative-ON patients may differ, and such differences will not only help to distinguish between the two conditions but also elucidate the underlying mechanisms of visual network damage. In our study, we used RS-fMRI with graph theory analysis to investigate the static and dynamic functional network changes resulting from visual impairment. Changes in topological metrics and their variance were found in MOG-ON and seronegative-ON, which may be able to discriminate the two entities. Although MOG-ON patients did not present different functional connections (edges) from seronegative-ON patients, MOG-ON patients presented more enhanced connections than HCs did. Our findings may be helpful in understanding the underlying changes in the neural mechanisms of ON.

In our study, compared with HCs, both MOG-ON and seronegative-ON patients presented significantly decreases in σ, whereas increases in the Lp were identified only in MOG-ON patients. The human brain functional network is defined as a complex and interconnected system with various topological features, including the small-world network. The small-world network, characterized by high efficiency at low cost and highly connected nodes, which implies both a high Cp and low Lp, may represent the balance between functional integration and segregation (Rubinov and Sporns, 2010; Yu et al., 2018). The most pronounced symptom in patients with ON, regardless of subtype, is significant vision loss that interferes with daily work and study. Damage to the optic nerve leads to disturbances in the transmission and subsequent processing of visual information, which in turn negatively impacts the efficiency of information transfer throughout the brain network (Zhou et al., 2024). According to our results, the reduced σ in ON patients precisely reflects the attenuation of small-world properties. In addition, regarding the dynamic global properties, ON patients presented greater variances than HCs. These also indicated the disturbance and vulnerability of brain functional networks in ON patients. Moreover, changes in the static and dynamic parameters were not identical; for example, Eg and Eloc did not show a significant difference in the static network, but its variance differed in the dynamic network. Thus, we suggest that the dynamic properties may provide additional valuable information and that the combination of static and dynamic properties will be more sensitive in reflecting changes in the efficiency of information transfer.

However, given the acute visual loss and the known plasticity of the visual system, the aforementioned changes compared with HCs were all within our expectations. Interestingly, there was a distinct tendency toward increased static and dynamic global properties and decreased σ in MOG-ON patients compared with those in seronegative-ON patients, which could be helpful in the differential diagnosis. Differentiation between these two entities is particularly important during the first episode of ON when the antibody status is unknown. As previously reported (Darakdjian et al., 2022; Kraker and Chen, 2023; Jeyakumar et al., 2024), acute ON associated with MOGAD was more likely to present with bilaterally and longitudinally extensive optic nerve involvement. In our study, MOG-ON patients had a trend toward more bilateral morbidity (30.4% vs. 10.0%), although there was no statistical difference. In MOG-ON, we believe that the more fragile small-world properties and weaker information transfer efficiency for interconnected regions shown on RS-fMRI are logically consistent with the larger area and longer segment optic nerve lesions shown on conventional MRI. This suggests that metrics of functional network topology may help discriminate between MOG-ON and seronegative-ON. Regarding the underlying pathophysiological mechanisms, pathological changes caused by MOG antibodies may directly lead to abnormalities in the transmission and communication of neuronal information, affecting a wide range of sites, compared to the serum-negative state. Additionally, MOG antibodies are also likely to exacerbate optic nerve injury via antibody-dependent cell-mediated phagocytosis (ADCP) and cytotoxicity (ADCC), as well as complement-mediated cytotoxicity and antigen presentation (Peschl et al., 2017; Spadaro et al., 2018), which may be related to the involvement of bilateral optic nerves in MOG-ON. This, in turn, exacerbates the abnormalities of MOG-ON. However, importantly, whether changes in the brain functional network are more sensitive and appear earlier than those of conventional MRI remains unknown and may require further longitudinal studies.

In addition to global properties, significant changes in local properties were also found in the VN, temporal lobes and prefrontal cortex (PFC). In the patient groups (either MOG-ON or seronegative-ON), increased Ne, Dc and Bc values were found in the CAL, CUN, LING, FFG and MTG, which are regions associated with the primary and secondary visual cortex (Kastner and Ungerleider, 2000; Galletti and Fattori, 2018; Zhou et al., 2024). In the occipital lobe, the CAL is thought to be the primary visual cortex; the CUN and LING are also key to basic and high-level visual processing in the visual field, visual memory and color vision (Kastner and Ungerleider, 2000; Galletti and Fattori, 2018). In addition, the FFG and MTG in the temporal lobes are considered to be parts of the ventral visual pathways of the VN, comprising high-level visual processes such as face perception and reading (Weiner and Zilles, 2016). The VN is the center for processing and integrating visual information transmitted via the optic nerve. To read and recognize objects, the brain regions located in either the primary or secondary VN can strengthen their intercommunication and visual information transfer efficiency to form a visual function compensatory mechanism. We attribute the increases in local properties of these regions to this mechanism. Notably, some VN-related regions, CAL (decreased Bc), LING (decreased NCp), SOG and FFG (decreased Ne), also presented decreased local properties, which contradicts our speculation above. This finding may be related to the severity of visual impairment and the strength of compensatory mechanisms, as well as differences in the sensitivity of the response among local properties. The HIP, located in the ventral pathway of the VN and associated with memory (Conway, 2018), also exhibited a decrease in the nodal property (Ne), which could be attributed to impaired memory efficiency due to visual impairment. In addition to regions with reduced nodal properties, we found that, in contrast to the ventral pathway of the VN, nodal properties (Ne, Dc, Bc, and NCp) were generally decreased in the parietal lobes and PFC (ORBsup, ORBsupmed, ACG, PoCG), regions located in the dorsal visual pathways and their associated regions (Galletti and Fattori, 2018). The ventral pathway of the VN is involved in graphical vision and object shape perception, whereas the dorsal pathway is associated with the processing of spatial position and related motion information (Conway, 2018; Galletti and Fattori, 2018). We speculate that the efficiency of advanced processing of visual information regarding the spatial position of objects may be impaired during the course of the disease and that compensatory mechanisms have not yet been developed. Furthermore, the PFC is linked to cognitive and emotional processes (Carlén, 2017). Impaired visual function may detrimentally affect these functions indirectly, including negative effects on emotional state, executive functioning, and decision-making.

With respect to the dynamic analysis, the variance in certain nodal properties of patient groups increased in the PHG, CUN, MOG, FFG, IOG, PCL and ITG compared to the HCs. These regions are also associated with the VN, including its ventral and dorsal pathways. This finding may also indicate the vulnerability of node functions, and comparing dynamic analysis with static methods may provide additional information (increased variance in the PHG, IOG and ITG). Furthermore, in the same way that global metrics could be used for differential diagnosis, local metrics in MOG-ON, i.e., decreased Ne (HIP, SOG and FFG), increased Dc (CUN), increased Bc (FFG) and increased Ne_var., Bc_var., and NLe_var (PHG, CUN, and MOG), could be compared with those in seronegative-ON. More drastic changes in information transfer efficiency, which may be due to more severe damage to the optic nerve in MOG-ON patients, may help to differentiate MOG-ON from seronegative-ON in terms of neural mechanisms.

In the edge analysis, an enhanced subnetwork (24 nodes and 28 edges) was identified in MOG-ON patients compared with HCs, mainly in the occipital, frontal and temporal lobes. The nodes were generally associated with VN function and were distributed across the VN and its ventral and dorsal pathways. Enhanced functional connectivity between the VN and other brain regions has also been reported in some optic nerve-related pathologies (Zhou et al., 2024), and we hypothesize that increased intercommunication between these nodes associated with either the primary or secondary VN may act as a compensatory mechanism for reduced visual acuity and visual field. Unfortunately, functional connectivity cannot be used for differentiation because connections did not differ between MOG-ON and seronegative-ON patients.

Correlation analysis showed that the functional topological organization metrics did not correspond to the severity of clinical symptoms or OCT measurements. Possibly due to the presence of compensatory mechanisms of brain function, the change in functional metrics may not fluctuate as much as ophthalmologic examinations, but may be a relatively stable change.

The static and dynamic metrics at the global and local levels can serve as biomarkers of brain network changes in MOG-ON and seronegative-ON patients. When the diagnostic abilities of the models for differentiating MOG-ON from seronegative-ON were compared, ROC curve analysis suggested that both the static and dynamic models had acceptable AUCs. The discriminative power of the combination of static and dynamic networks was greatest, reflecting that combined model was more clinically valuable than the single model. Thus, static and dynamic brain network topology analysis based on RS-fMRI is particularly useful for differentiation in ON patients, especially during the first attack when the serum antibody status cannot be obtained quickly.

This study had several limitations. First, the limited number of patients from a single center may bias the study results. A larger cohort of subgroups, including new subgroups, such as NMO-related ON, is still needed to reduce the risk of bias. Second, although we enrolled first-episode patients without cranial or spinal demyelinating lesions in this study, some patients in the seronegative-ON group may progress to MS, which may have an unknown effect on our results. Therefore, we are continuing to follow these patients and look forward to further longitudinal studies. Third, this work was a cross-sectional observational study. Thus, the order in which optic nerve lesions and functional brain changes occur cannot be assessed, although our findings in MOG-ON patients, who present with more severe abnormalities in information efficiency, appear to be consistent with the more extensive lesions in the optic nerves. Furthermore, the ability to discern causality between clinical symptoms and functional network changes is also limited. Longitudinal studies are still needed in the future. Fourth, to fully exploit the value of dynamic functional networks, variability matrix comparisons and cluster analysis will also be used in further in-depth studies.

In conclusion, the present study demonstrated the abnormal topological organization of static and dynamic brain functional networks in ON patients. Furthermore, disturbed topological features may help to better understand the neural mechanisms of ON and may serve as biomarkers to help differentiate MOG-ON from seronegative-ON.

## Data Availability

The raw data supporting the conclusions of this article will be made available by the authors, without undue reservation.

## Footnotes

^1^ https://www.fil.ion.ucl.ac.uk/spm/software/spm12

^2^ http://www.nitrc.org/projects/gretna/

^3^ http://restfmri.net/forum/DynamicBC

^4^ https://www.nitrc.org/projects/bnv/

^5^ http://www.nitrc.org/projects/nbs/
